# Volume replacement strategies do not impair the binding of dabigatran to idarucizumab: Porcine model of hemodilution

**DOI:** 10.1371/journal.pone.0209350

**Published:** 2019-01-07

**Authors:** Oliver Grottke, Joanne van Ryn, Christian Zentai, Guanfa Gan, Markus Honickel, Rolf Rossaint, Hugo ten Cate, Henri M. H. Spronk

**Affiliations:** 1 Department of Anesthesiology, RWTH Aachen University Hospital, Aachen, Germany; 2 Department of CardioMetabolic Diseases Research, Boehringer Ingelheim GmbH & Co. KG, Biberach, Germany; 3 Boehringer Ingelheim Pharmaceuticals, Inc., Ridgefield, Connecticut, United States of America; 4 Laboratory for Clinical Thrombosis and Haemostasis, Department of Internal Medicine, Cardiovascular Research Institute Maastricht, Maastricht University Medical Center, Maastricht, The Netherlands; Leiden University Medical Center, NETHERLANDS

## Abstract

**Background:**

Idarucizumab is a humanized Fab fragment that specifically reverses dabigatran anticoagulation. In trauma, volume expanders are used for resuscitation to compensate for blood loss and hemorrhagic shock, but it is unknown whether volume expanders influence the binding of dabigatran to its antidote. Using a porcine dilutional coagulopathy model, this study investigated whether volume replacement strategies affect binding of dabigatran to idarucizumab.

**Methods:**

Twenty-five male pigs were treated orally with dabigatran etexilate (30 mg/kg bid) for 3 days. The following day, animals were anesthetized, infused with dabigatran (total dose 0.645 mg/kg) to achieve supratherapeutic concentrations, and randomized 1:1:1:1:1 (n = 5 per group) to control (no hemodilution) or hemodilution where ~50% of blood volume was substituted with Ringer’s solution, 6% hydroxyethyl starch 130/0.4, 6% hydroxyethyl starch 200/0.5 or 4% gelatin. Idarucizumab was then administered intravenously (30 mg/kg) and serial blood samples were taken for up to 24 hours to measure diluted thrombin time (corresponding with dabigatran activity), total dabigatran (bound to antidote and free drug) and a panel of coagulation parameters.

**Results:**

Mean plasma dabigatran levels were 617 ± 16 ng/mL after infusion and 600 ± 114 ng/mL after ~50% hemodilution with no significant differences between groups. Following treatment with idarucizumab, plasma concentrations of unbound dabigatran decreased markedly, with similar reductions in all groups. Dabigatran-induced prolongation of coagulation parameters was rapidly reversed in all groups.

**Conclusion:**

This study indicates that several volume expanders used for resuscitation in trauma do not interfere with the binding of idarucizumab to dabigatran.

## Introduction

Post-traumatic bleeding is a leading cause of mortality following trauma [1]. Coagulation abnormalities are common in trauma patients and contribute significantly to morbidity and mortality. Causes of coagulopathy include blood loss, dilution and consumption of coagulation factors, hypothermia and activation of fibrinolysis [2]. Use of oral anticoagulants can exacerbate trauma-induced coagulopathy and increase blood loss [3].

Idarucizumab, a humanized monoclonal antibody fragment specific to dabigatran, is approved for reversing the anticoagulant activity of dabigatran in patients with uncontrolled bleeding or requiring emergency procedures [4]. By binding to dabigatran with a specificity ~350 times greater than the binding of dabigatran to thrombin, idarucizumab rapidly inactivates dabigatran in plasma, as demonstrated by assays such as activated partial thromboplastin time (aPTT), ecarin clotting time (ECT) and diluted thrombin time (dTT) [5]. Idarucizumab binds to both dabigatran and its active metabolites (glucuronides), forming stable complexes. It does not bind endogenous thrombin substrates, activate coagulation factors or platelets, nor does it elevate thrombin generation in volunteers [6,7]. Therefore, in the absence of dabigatran, it has no effect on coagulation status. Interim analyses of the phase III RE-VERSE AD study showed that idarucizumab immediately reversed dabigatran-induced anticoagulation in a heterogeneous patient population [4]. Further, in a lethal preclinical trauma model under dabigatran anticoagulation, idarucizumab significantly reduced blood loss [8].

In patients experiencing trauma or severe hemorrhage, volume expanders may be used to maintain circulation, oxygen delivery and avoid severe shock. Initial fluid resuscitation generally involves the use of crystalloids, while colloid volume expanders such as hydroxyethlystarch (HES) and 4% gelatin are recommended for persistent hemorrhagic shock [1]. Resuscitation with large volumes of crystalloids has been associated with tissue edema, and increased incidence of abdominal compartment syndrome [2]. Compared to crystalloids, colloids can induce more rapid and persistent plasma expansion because of a larger increase in oncotic pressure, and thus achieve circulatory goals more quickly. However, there is no survival benefit when colloids are administered and HES has been associated with a risk of kidney injury and mortality; in the EU, the use of HES is currently restricted to severe shock refractory to crystalloid resuscitation [9,10].

Dabigatran-treated patients requiring emergency procedures and receiving idarucizumab may also require concurrent volume replacement. It is unknown whether volume expanders might influence the binding of idarucizumab to dabigatran, potentially reducing its ability to reverse the anticoagulant effect of dabigatran. The present study was performed to investigate the effects of frequently used volume expanders on the binding of idarucizumab to dabigatran over 24 hours in a porcine model of dilutional coagulopathy with 50% blood loss.

## Materials and methods

### Ethics and anesthesia

All experimental procedures were approved by the local animal care committee (Landesamt für Natur, Umwelt und Verbraucherschutz Nordrhein-Westfalen, Recklinghausen, Germany) and conducted in accordance with the German Animal Protection Act. Twenty-five male German Landrace pigs weighing 50 *±* 5 kg (mean *±* standard deviation) were housed in ventilated rooms and acclimatized for a minimum of 7 days before oral dosing with dabigatran etexilate twice-daily for three consecutive days (30 mg/kg, 150 mg Pradaxa capsules). The final dose was administered 12 hours before hemodilution. Animals were fasted after the final dose and water was provided ad libitum. On day 4, anesthesia was induced as described previously [11,12,13].

### Preparation, hemodilution and dabigatran infusion

For surgical preparation, animals received an intramuscular injection of 4 mg/kg azaperone (Stresnil; Janssen, Neuss, Germany) and 0.1 mg/kg atropine (atropine sulphate; B Braun, Melsungen, Germany). Catheters for blood sampling and infusions were placed in the right carotid artery and left jugular vein and a 90-minute infusion of dabigatran (10 mg/mL solution: 0.77 mg/kg/h for 30 minutes and 0.26 mg/kg/h for 60 minutes) was given to achieve consistent plasma concentrations. Anesthesia was induced by intravenous injection of 3 mg/kg propofol (Disoprivan; AstraZeneca, Wedel, Germany), and this was followed by orotracheal intubation. The animals were ventilated with a tidal volume of 8 mL/kg and 16–22 breaths/minute (Cato ventilator; Draeger, Luebeck, Germany) to maintain end-tidal CO_2_ at 36–42 mmHg. Anesthesia was maintained with isoflurane (Forane; Abbott Laboratories Inc., Abbott Park, IL, USA) at an end-tidal concentration of 1%, and continuous infusion of fentanyl (Janssen, Neuss, Germany) at 3–4 μg/kg/h.

Animals were randomly allocated (n = 5 per group) to control (no hemodilution) or hemodilution with one of four volume expanders: balanced Ringer’s solution (Sterofundin, B.Braun, Melsungen, Germany), 6% HES 130/0.4 (Voluven; Fresenius Kabi, Bad Homburg, Germany), 6% HES 200/0.5 (HAES2; Fresenius Kabi, Bad Homburg, Germany), or 4% gelatin (Gelafundin; B.Braun, Melsungen, Germany). In the hemodilution groups, ~50% of the animal’s estimated blood volume (65 mL/kg body weight) was withdrawn at a rate of 100 mL/min following the dabigatran infusion. The appropriate volume expander was then administered over 30 minutes. The total infusion volume of Ringer’s solution was the same as the volume of blood withdrawn, while the dose for all three colloids was 25 mL/kg.

### Idarucizumab treatment and follow up

Idarucizumab (44 mg/mL solution; dose, 30 mg/kg) was administered as an intravenous bolus to all groups immediately following hemodilution. Four hours after treatment with idarucizumab, animals were extubated and allowed to recover. Carprofen (Rimadyl, Pfizer, Berlin, 4 mg/kg) was given i.m. for postoperative pain relief. At the end of a 24-hour observation period, animals were euthanized with an overdose of pentobarbital.

### Blood sampling and analytical methods

Blood samples were taken prior to any treatment (baseline), after i.v. dabigatran, after hemodilution and at 5 minutes, 15 minutes and 3, 12 and 24 hours after idarucizumab dosing. Blood gases were assessed in heparinized blood samples using an ABL700 blood gas analyzer (Radiometer, Copenhagen, Denmark). A standard hematology analyzer (MEK-6108, Nihon Kohden, Rosbach, Germany) was used for measurement of hemoglobin, erythrocytes, white cell count and platelet count in samples that had been transferred to potassium-EDTA-anticoagulant tubes. Sodium citrate (Sarstedt, Nümbrecht, Germany; 3.2%) and EDTA-anticoagulant (Sarstedt) were added to blood samples for coagulation assays and measurements of drug concentrations, respectively, before centrifugation to obtain cell free plasma; the plasma samples were stored at -80°C prior to assessment. Standard laboratory methods were used to measure aPTT (CK Prest reagent [Diagnostica Stago, Asnierès sur Seine, France]; BCS XP analyzer [Siemens, Erlangen, Germany]), dTT (Hemoclot Direct Thrombin Inhibitor assay [Hyphen BioMed, Neuville sur-Oise, France]; BCS XP analyzer [Siemens, Erlangen, Germany]), fibrinogen concentration (Thrombin reagent; CA7000 analyzer [both from Siemens, Erlangen, Germany]) and D-dimer levels (Innovance D-dimer assay; BCS XP analyzer [both from Siemens, Erlangen, Germany]). Non-anticoagulated whole blood was used for measurement of activated clotting time (ACT), using celite cartridges and an i-STAT point-of-care device (Abbott, Princeton, NJ, USA). Thromboelastometry was performed with commercially available reagents (ex-tem and in-tem) in citrated whole blood on a ROTEM device (Tem international GmbH, Munich, Germany).

### Dabigatran and idarucizumab: Plasma levels

Levels of active, unbound dabigatran in plasma were measured using the dTT assay in citrated plasma [14]. Total dabigatran (including active unbound dabigatran and its active glucuronide metabolites, inactive idarucizumab-bound and plasma protein-bound dabigatran) was assessed at Nuvisan GmbH & Co. KG (Neu-Ulm, Germany) by validated liquid chromatography-mass spectrometry (LC-MS/MS) [15,16].

Idarucizumab concentrations in plasma were assessed at Boehringer Ingelheim (Ridgefield, CT, USA) using an enzyme-linked immunosorbent assay (ELISA) for porcine plasma. Antihuman IgG antibody (2 μg/mL) was applied overnight to microtitre plates; this was subsequently incubated with calibration standards, anchor points, blank plasma, quality controls, and samples. Horseradish peroxidase (HRP)-conjugated sheep antihuman IgG antibody (0.5 μg/mL) was added, and tetramethylbenzidine was used to detect bound HRP-conjugate (a plate reader was used to take readings). The lower limit of quantification for idarucizumab was 150 ng/mL (3.14 nM).

### Pharmacokinetic assessment

Non-compartmental analysis of plasma idarucizumab, total dabigatran, and active unbound dabigatran concentration–time data was performed using Kinetica Version 4.4.1 software (Thermo Scientific, Philadelphia, PA, USA). Standard methods were used to calculate the area under the plasma concentration–time curve (AUC) over 24 h (AUC_0-24_), peak plasma concentration (C_max_), clearance (CL), and terminal elimination half-life (t_½_). Plasma concentrations immediately before idarucizumab dosing were considered as the time zero values.

### Statistical analysis

As this was an explorative study, no power analysis was performed. Data for each treatment group were reported with descriptive statistics. Due to the small group size (n = 5/group), data were treated as non-normally distributed and results were presented as median ± interquartile range (IQR), unless indicated otherwise. Differences between the control group and each volume replacement group were assessed by pairwise between-group comparisons with Wilcoxon-Mann-Whitney tests followed by Bonferroni adjustment. SPSS 23 (SPSS, Chicago, IL, USA) was used to carry out statistical tests with two-tailed methodology; the level of significance was p<0.05. All graphs were created using GraphPad Prism (v6.0h, GraphPad Software Inc., San Diego, CA, USA).

## Results

**Fig 1:**
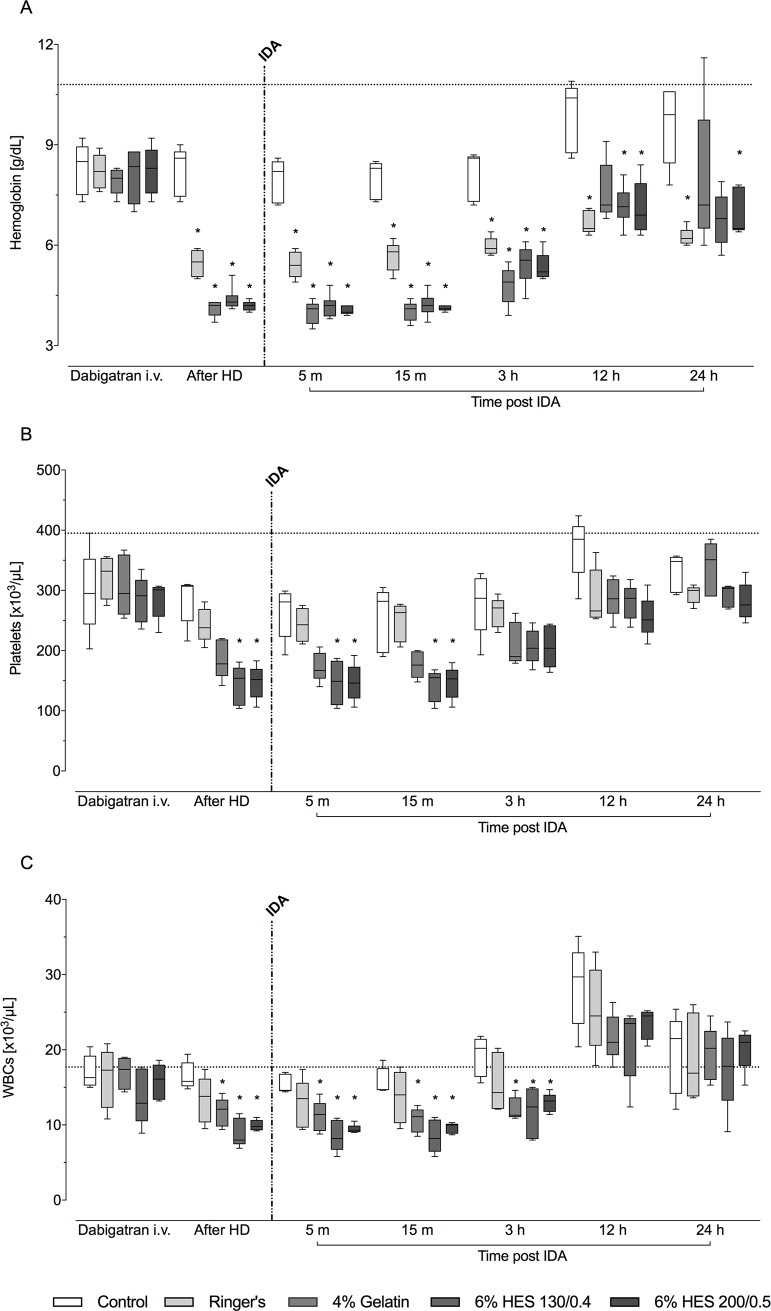
Hemoglobin levels (A), platelet (B) and white blood cell counts (C) after intravenous dabigatran, after hemodilution, and after idarucizumab injection. Dotted black horizontal lines indicate median baseline values (n = 25). HD: hemodilution; IDA: idarucizumab; WBCs: white blood cells. Data are shown as median values; boxes extend from 25^th^ to 75^th^ percentiles and whiskers show minimum and maximum values (n = 5/group). *P<0.05 vs. control group.

Prior to oral dabigatran treatment, all assessments including coagulation parameters and blood cell counts, were similar across the five study groups (Table 1). Hemodilution and volume replacement over 60 minutes led to a 40–50% decrease in hemoglobin levels as compared with control (p<0.05) (Fig 1A). Subsequently, hemoglobin levels increased slowly throughout the 24-hour observation period in the hemodilution groups, but statistically significant differences versus control were maintained. The hemoglobin level remained stable over time in the control group. Platelet and white blood cell counts followed the same pattern as observed for hemoglobin, although significant differences from controls in the hemodilution groups were not seen after 3 hours post-idarucizumab (Fig 1B and 1C).

**Table 1:** Baseline parameters in the different groups prior to oral dabigatran treatment.

Parameter	Control	Ringer’s	4% Gelatin	6% HES 130/0.4	6% HES 200/0.5
Platelets [x10^3^/μL]	390 [349–462]	388 [342–513]	393 [369–454]	383 [345–481]	422 [346–436]
Hemoglobin [g/dL]	10.9 [9.1–11.3]	10.8 [10.2–11.2]	10.0 [9.2–11.3]	10.8 [9.8–11.4]	11.1 [10.4–11.3]
Fibrinogen [g/L]	1.8 [1.3–2.3]	1.6 [1.5–1.8]	1.6 [1.5–2.0]	1.6 [1.4–1.8]	1.6 [1.3–1.9]
WBC [x10^3^/μL]	19.5 [13.7–24.5]	19.2 [8.4–21.6]	17.4 [15.0–21.4]	16.5 [15.3–17.7]	17.5 [13.6–22.1]
aPTT [sec]	15 [14–16]	15 [14–15]	14 [14–16]	17 [15–18]	16 [14–16]
D-Dimer [ng/mL]	750 [400–1040]	520 [350–650]	680 [470–890]	1115 [360–2810]	370 [170–650]
ACT [sec]	87 [72–110]	110 [99–114]	99 [76–118]	99 [76–106]	103 [76–106]

Values are shown as median [IQR], n = 5 animals per group, no significant differences between groups

ACT, activated clotting time; aPTT, activated partial thromboplastin time; HES, hydroxyethyl starch; WBC, white blood cells

### Plasma dabigatran concentrations

**Fig 2:**
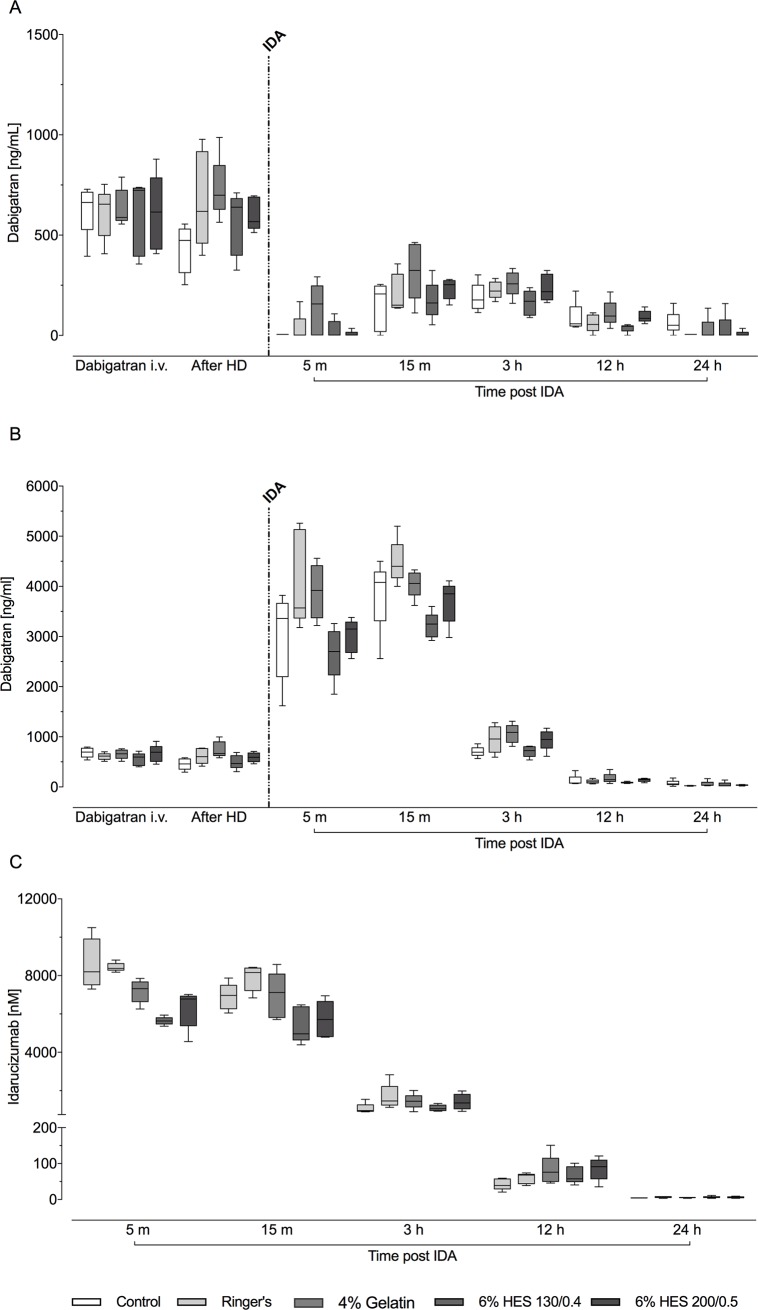
Plasma concentration of active dabigatran assessed by means of the diluted thrombin time (dTT) assay (unbound dabigatran, A); plasma concentration of total dabigatran assessed by LC-MS/MS (B); and plasma idarucizumab levels assessed by ELISA (C) after intravenous dabigatran, after hemodilution, and after idarucizumab injection. HD: hemodilution; IDA: idarucizumab. Data are shown as median values; boxes extend from 25^th^ to 75^th^ percentiles and whiskers show minimum and maximum values (n = 5/group).

Dabigatran etexilate dosed orally (30 mg/kg twice-daily) for 3 days resulted in a median trough plasma dabigatran concentration of 352 (IQR, 47–795) ng/mL across the treatment groups (data not shown). After the infusion of dabigatran and before hemodilution, the plasma concentration of dabigatran had increased to a median of 611 (596–636) ng/mL, with no significant differences between the groups (Fig 2A).

In the control group, the plasma dabigatran concentration was initially 663 (395–729) ng/mL post-infusion, decreasing to 474 (253–555) ng/mL after 1 hour, representing normal clearance of the drug (Fig 2A). Following hemodilution and volume replacement, plasma dabigatran concentrations were comparable among the four treatment groups (Ringer’s, 6% HES 130/0.4, 6% HES 200/0.5 and 4% gelatin) prior to idarucizumab administration.

Plasma concentrations of unbound dabigatran decreased in all groups within 5 minutes of idarucizumab administration, to 0 (0–292) ng/mL (Fig 2A). There was a slight increase over the following 10 minutes to 211 (0–463) ng/mL, representing a partial return of anticoagulation. Levels of unbound dabigatran decreased slowly after the 15-minute time point, reaching 0 (0–160) ng/mL after 24 hours. Changes over time in plasma dabigatran concentrations were similar in all four volume replacement groups and the control group.

Hemodilution with any of the four volume expanders did not change the total dabigatran concentrations in pigs (Fig 2B). Immediately following idarucizumab administration, ~5-8-fold increases in plasma concentrations of total dabigatran were seen (Fig 2B). Thus, the majority of dabigatran molecules were bound to idarucizumab and inactive. A consistent pattern was evident across all volume replacement strategies; slight differences between the groups were attributable to variations between animals in dabigatran and idarucizumab concentrations.

**Fig 3:**
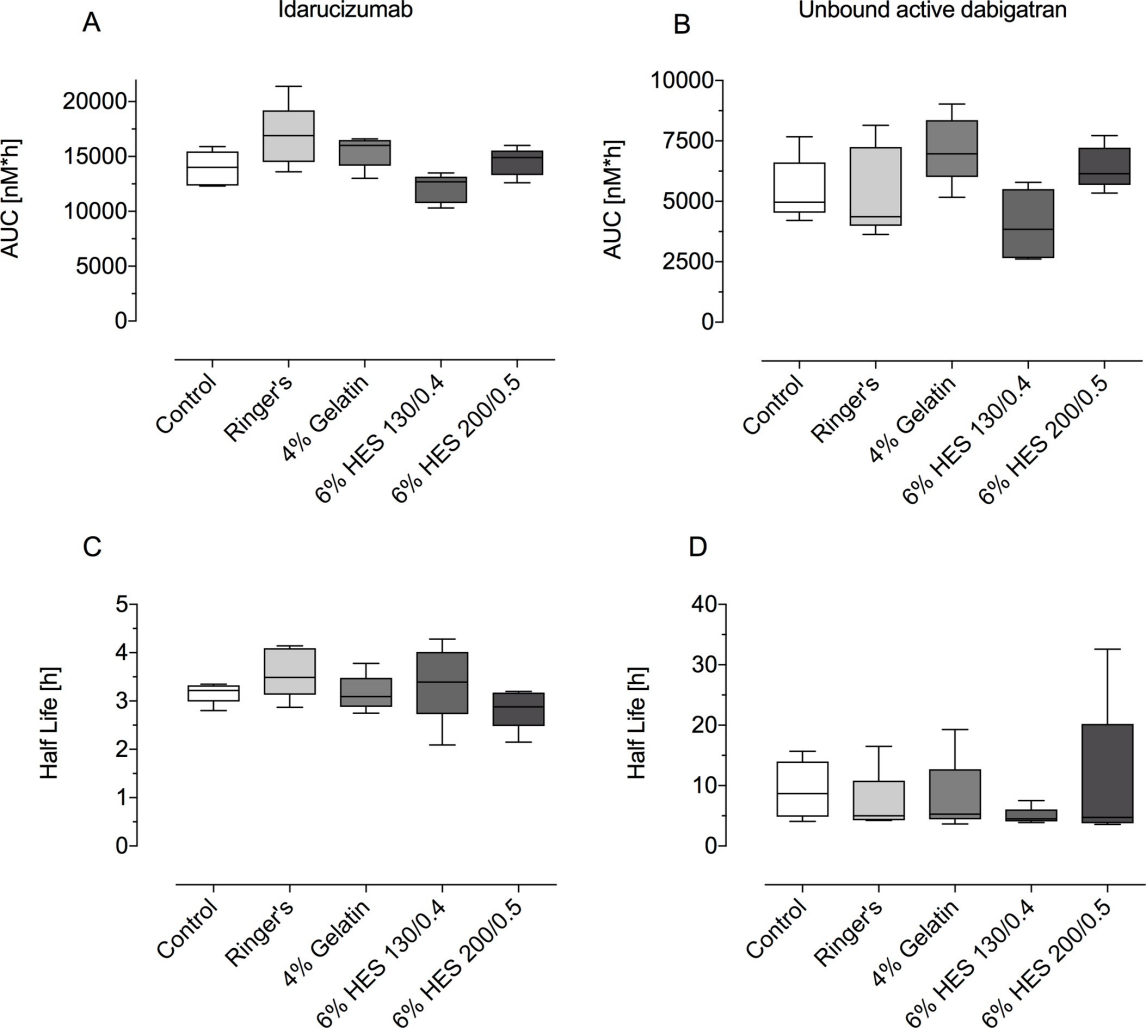
Comparison of AUC_0-24_ and half-life of idarucizumab (A, C) and unbound dabigatran (B, D) across the treatment groups. Data are shown as median values; boxes extend from 25^th^ to 75^th^ percentiles and whiskers show minimum and maximum values (n = 5/group).

There were no significant differences in the pharmacokinetic parameters of total and unbound active dabigatran (Fig 3; S1 and S2 Tables) between the control group and the four treatment groups. Unbound active dabigatran exposure (AUC_0-24_) was similar in all of the volume expander groups with no significant differences versus control (Fig 3B).

### Plasma idarucizumab concentrations

Plasma idarucizumab concentrations were highest at 5 minutes post-injection, the first post-dose time point. Median concentrations at this time ranged between 8200 (7300–10500) nM in the control group and 5630 (5360–5940) nM in the 6% HES 130/0.4 group (Fig 2C). Idarucizumab levels decreased over time in a similar manner in all five study groups. There were no substantial differences in the concentration–time profiles and pharmacokinetic parameters of idarucizumab between the five groups (Fig 2C; S3 Table). The terminal half-life, AUC and clearance of idarucizumab in the control group were similar to those in the groups treated with different volume expanders (S3 Table).

### Coagulation parameters

**Fig 4:**
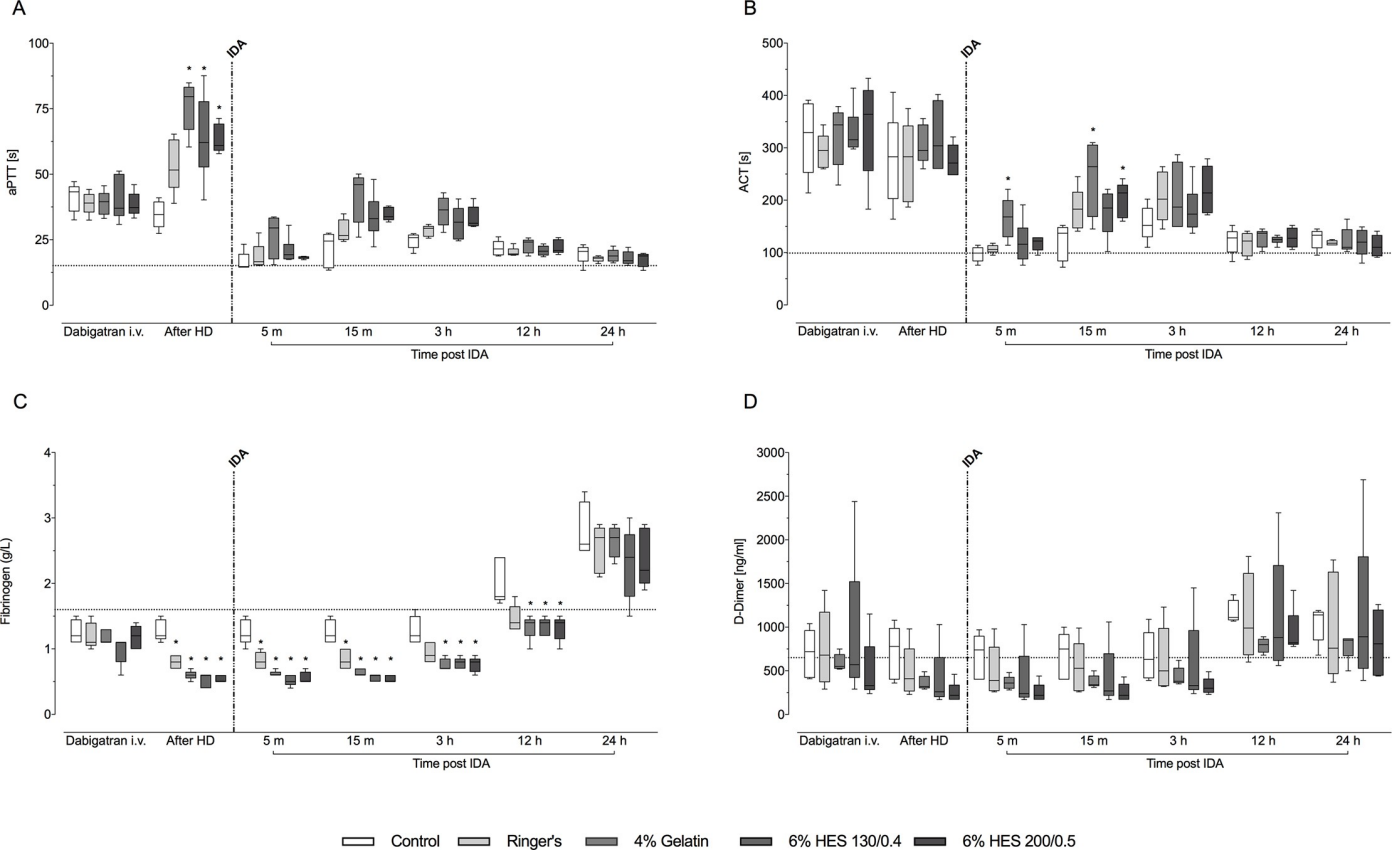
Activated partial thromboplastin time (aPTT; A), activated clotting time (ACT; B), plasma fibrinogen concentration (C), and levels of D-dimers (D) after intravenous dabigatran, after hemodilution, and after idarucizumab injection. Dotted black horizontal lines indicate median baseline values (n = 25). HD: hemodilution; IDA: idarucizumab. Data are shown as median values; boxes extend from 25^th^ to 75^th^ percentiles and whiskers show minimum and maximum values (n = 5/group). *P<0.05 vs. control group.

Dabigatran treatment prolonged the aPTT (all animals) from 15 (15–17) to 39 (39–41) seconds and the ACT from 101 (72–118) to 329 (183–433) seconds, representing increases of 2.7- to 3.3-fold from baseline (Fig 4A and 4B). Hemodilution prolonged aPTT further.

Treatment with idarucizumab promptly decreased aPTT and ACT, with values 5 minutes post-treatment that were close to baseline (Fig 4A and 4B). Only minor differences were observed between the different volume expanders. Slight increases in aPTT and ACT were seen at 15 minutes and 3 hours after idarucizumab treatment, reflecting the small increases in unbound dabigatran levels. Plasma fibrinogen levels were significantly lower after hemodilution as compared to control (Fig 4C), and significant differences versus control were sustained in the 4% gelatin, 6% HES 130/0.4 and 6% HES 200/0.5 groups until 12 hours after idarucizumab treatment. D-dimer levels remained close to baseline throughout the study, with small differences between groups (Fig 4B).

**Fig 5:**
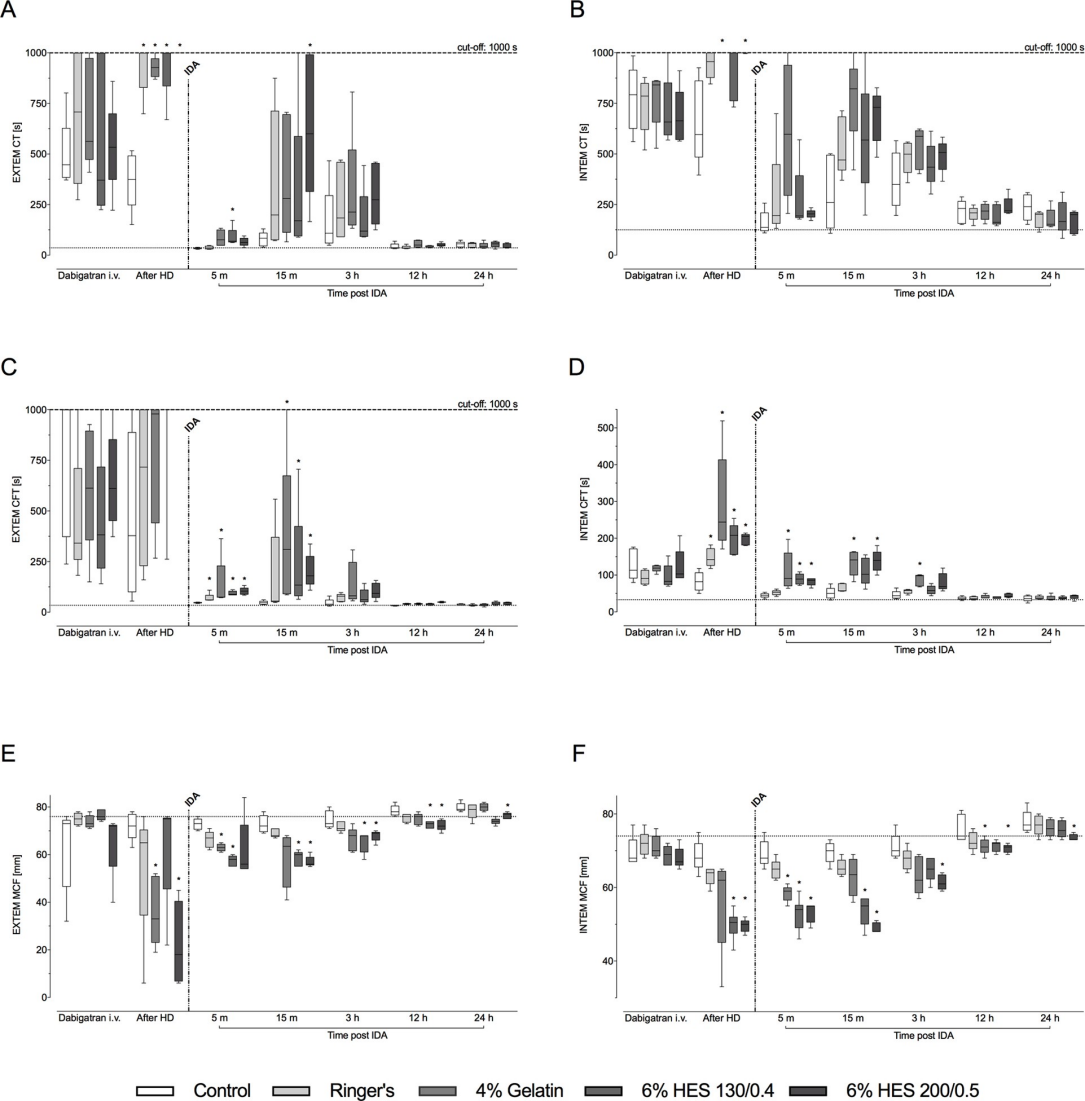
Clotting time (CT; A,B), clot formation time (CFT; C,D), and maximum clot firmness (MCF; E,F) after intravenous dabigatran, after hemodilution, and after idarucizumab injection. Left panel showing the results of the EXTEM test, right panel those of the INTEM test. Dashed red horizontal lines indicate a cut-off at 1000 s. Dotted black horizontal lines indicate median baseline values (n = 25). HD: hemodilution; IDA: idarucizumab. Data are shown as median values; boxes extend from 25^th^ to 75^th^ percentiles and whiskers show minimum and maximum values (n = 5/group). *P<0.05 vs. control group.

Clotting time (CT) and clot formation time (CFT) in thromboelastometry (EXTEM) were initially prolonged by dabigatran, with median values of 572 (494–691) and 623 (450–749) seconds, compared to baseline values of 37 (34–41) and 34 (33–37) seconds, respectively (Fig 5A–5C). Similar prolongations were also observed in the INTEM test (Fig 5B–5D). Hemodilution caused further deterioration in all of these thromboelastometry variables (Fig 5). Kinetic parameters of EXTEM (CT, CFT), and—to a lesser extent—of INTEM, were immediately returned to levels close to baseline after administration of idarucizumab. At 15 minutes and 3 hours, a degree of rebound was observed, coinciding with the small increases in levels of unbound dabigatran. CT and CFT in the EXTEM and INTEM assays were close to baseline at 12 and 24 hours post-idarucizumab. Maximum clot firmness (MCF) in both the EXTEM and INTEM assays was reduced following hemodilution, but then increased gradually to reach levels close to baseline at 24 hours. There was some variation between the four hemodilution groups, with slight trends towards greater decreases from baseline in the two HES groups. A slight increase over time in MCF was apparent in the control group, in both the EXTEM and INTEM assays–but in comparison with the hemodilution groups, changes over time in the control group were small.

## Discussion

This study showed that hemodilution and volume resuscitation had no impact on the concentration–time profiles or pharmacokinetic parameters of idarucizumab, total dabigatran or unbound active dabigatran. Furthermore, none of the four volume expanders investigated in the study had a significant impact on the reversal of dabigatran’s anticoagulant activity by idarucizumab, as shown by the lack of significant differences in unbound dabigatran between the resuscitation groups and the control group without hemodilution. Minor differences observed between the volume expanders are attributable to their direct effects rather than effects on the ability of idarucizumab to bind and inactivate dabigatran.

The idarucizumab dose used in this study was not intended to completely reverse the anticoagulant activity of dabigatran. Instead, the study was designed to determine whether the different volume expanders had any impact on dabigatran-idarucizumab binding. This single dose of idarucizumab was able to inhibit ~25% of the total dabigatran on a molar basis. Since dabigatran-idarucizumab binding in the presence of hemodilution was not affected at this dose level, and it has been demonstrated in studies that idarucizumab has no effect on any other proteins or small molecules, other than dabigatran [5], it is not expected that different outcomes would be obtained with different dose levels. The presence of residual anticoagulant activity of dabigatran after reversal allowed more sensitive assessment of any potential interactions between volume expanders and the effects of idarucizumab.

A return of dabigatran anticoagulant activity within ~15 min following administration of idarucizumab was observed. This is explained by redistribution of dabigatran from extracellular space in tissues into the blood compartment as idarucizumab binds dabigatran in blood in a 1:1 stoichiometric manner, thereby reducing the plasma concentration of the active dabigatran. Preclinical studies have shown that idarucizumab binds dabigatran with a high binding affinity of 2.1 pM, a very fast on-rate and a slow off-rate resulting in immediate, almost irreversible binding. The effectiveness of idarucizumab in reversing dabigatran-induced coagulopathy depends on whether the dose of idarucizumab is sufficient (i.e. equimolar) to inhibit dabigatran [5]. In humans, the approved dose of idarucizumab (5 g) was calculated to inhibit plasma levels up to the 99th percentile of peak dabigatran levels measured in the RE-LY study [17], with the intention of achieving reversal in the majority of patients undergoing dabigatran therapy. Interim analysis of the RE-VERSE AD phase III clinical trial confirmed the effectiveness of this dose [4]. Nonetheless, a repeat dose of idarucizumab may be required in a small proportion of patients, such as those with high plasma levels of dabigatran related to acute renal impairment [18]. In the present study, the small differences in plasma dabigatran concentrations before and after hemodilution are likely due to redistribution of dabigatran. There was no apparent difference between the five study groups in the clearance of idarucizumab, indicating that there were no nonspecific interactions of idarucizumab with any of the resuscitation methods tested.

There were some differences in coagulation assay results according to the type of volume expander used. This could be related to direct effects on coagulation–it is known that coagulation assays are affected to differing degrees by different volume expanders [9,19]. For both HES 130/0.4 and 200/0.5 solutions, reduced clot strength in viscoelastic tests has been reported previously [19,20,21,22]. Despite the differences, rapid neutralization of dabigatran anticoagulant activity after the addition of idarucizumab was evident in all of the study groups and in all of the assays. Of the different coagulation assays evaluated in this study, the dTT assay was least affected by hemodilution. As previously demonstrated, the dTT assay response to dabigatran is linear over a large concentration range and is useful for determining active dabigatran plasma concentration based on clotting times [14]. The dTT assay is performed in plasma samples that are diluted with a final ratio of 1:24. Thus, most types of coagulopathy that relate to hemodilution have little effect on dTT results [23]. In contrast, the aPTT assay is performed using undiluted plasma, and the results are influenced by hemodilution at least as much as they are by dabigatran. Whole-blood assays, such as thromboelastometry and ACT, produced results that were consistent with those of the dTT assay. Thus, both point-of-care testing or conventional plasma-based coagulation assays could be used for qualitative assessment of dabigatran reversal with idarucizumab, even in the presence of hemodilution. However, none these tests provide specifically measure the effects of dabigatran and they should therefore be considered as surrogated markers.

We recognize that our study has some limitations. It was not feasible to include a group with hemodilution and a ‘placebo’ volume expander, meaning that it is difficult to study the effects of a volume expander only. Although some differences between groups reached statistical significance, the groups were small and this may have resulted in a lack of statistical power. In this and other investigations using similar porcine models, dilutional coagulopathy was induced through blood withdrawal and resuscitation fluid infusion. Such models may not include the complexities of real-world trauma patients, but one advantage is that the extent of hemodilution can be standardized [24]. The sequence of events is designed to mimic a clinical situation in which a trauma patient has been stabilized and hemostasis secured after initial hemorrhagic shock and resuscitation, and subsequent reversal of the dabigatran-induced coagulopathy is required. Human coagulation assay kits were used to measure coagulation assays; there may be some differences with porcine plasma.

In summary, this study demonstrates that volume expanders used in clinical practice for resuscitation do not interfere with the binding of dabigatran to its specific antidote, idarucizumab. The reversal of dabigatran anticoagulation can be assessed qualitatively with a range of surrogate markers including standard plasma-based coagulation assays, thromboelastometry or ACT, even in the presence of dilutional coagulopathy.

## Supporting information

S1 Table Summary pharmacokinetic parameters of total dabigatran determined by LC-MS/MS assay in pigs. Results are presented as mean (± SD), n = 5/group, unless stated otherwise.
(DOCX)

S2 Table Summary pharmacokinetic parameters of unbound active dabigatran determined by the dTT assay in pigs. Results are presented as mean (± SD), n = 5/group.
(DOCX)

S3 Table Summary pharmacokinetic parameters of idarucizumab in pigs after intravenous dosing. Results are presented as mean (± SD), n = 5/group, unless stated otherwise.
(DOCX)

S1 File Haemodilution all data. (XLSX)
