# Supplementary material for: Volume replacement strategies do not impair the binding of dabigatran to idarucizumab: Porcine model of hemodilution
Source: PLoS One. 2019 Jan 7;14(1):e0209350. doi: 10.1371/journal.pone.0209350 (PMC6322768; doi:10.1371/journal.pone.0209350)
Supplement: S1 Table — Results are presented as mean (± SD), n = 5/group, unless stated otherwise. (DOCX) [file pone.0209350.s001.docx]

**S1 Table** Summary pharmacokinetic parameters of total dabigatran determined by LC-MS/MS assay in pigs^a^

| **Group** | **C_max_ [nM]** | **t_max_ [h]**^b^ | **AUC_0-24_ [nM·h]** | **t_1/2_ [h]** |
| --- | --- | --- | --- | --- |
| Control | 8600 ± 715 | 0.25 (0.25-0.5) | 19200 ± 1940 | 5.30 ± 2.96 |
| Gelatin | 8830 ± 761 | 0.25 (0.083-0.25) | 24200 ± 2270 | 4.00 ± 1.01 |
| 6% HES 200/0.5 | 7830 ± 924 | 0.25 (0.25-0.25) | 21500 ± 2620 | 5.13 ± 0.826 |
| Ringer’s Solution | 9820 ± 1070 | 0.25 (0.083-0.25) | 22600 ± 3390 | 4.38 ± 0.208 |
| 6% HES 130/0.4 | 7050 ± 734 | 0.25 (0.25-0.25) | 16800 ± 1950 | 4.65 ± 1.06 |

^a^Results are presented as mean (± SD), n=5/group, unless stated otherwise.

^b^Median (range).

**AUC_0-24_** = area under the drug plasma concentration-time curve for time 0 to 24 h (time 0 was the time immediately before idarucizumab dosing); **C_max_** = maximum drug concentration in plasma; **t_1/2_** = terminal elimination half-life; **t_max_** = time to reach C_max_.
