# Supplementary material for: Volume replacement strategies do not impair the binding of dabigatran to idarucizumab: Porcine model of hemodilution
Source: PLoS One. 2019 Jan 7;14(1):e0209350. doi: 10.1371/journal.pone.0209350 (PMC6322768; doi:10.1371/journal.pone.0209350)
Supplement: S2 Table — Results are presented as mean (± SD), n = 5/group. (DOCX) [file pone.0209350.s002.docx]

S2 Table Summary pharmacokinetic parameters of unbound active dabigatran determined by the dTT assay in pigs^a^

| **Group** | **C_max_ [nM]** | **AUC_0-24_ [nM·h]** | **t_1/2_ [h]** |
| --- | --- | --- | --- |
| Control | 917 ± 257 | 5452 ± 1328 | 9.28 ± 4.77 |
| Gelatin | 1680 ± 278 | 7144 ± 1404 | 7.91 ± 6.43 |
| 6% HES 200/0.5 | 1280 ± 177 | 6390 ± 891 | 10.5 ± 12.4 |
| Ringer’s Solution | 1430 ± 508 | 5368 ± 1856 | 7.04 ± 5.30 |
| 6% HES 130/0.4 | 1150 ± 314 | 4034 ± 1445 | 4.96 ± 1.46 |

^a^Results are presented as mean (± SD), n=5/group.

**AUC_0-24_** = area under the drug plasma concentration-time curve for time 0 to 24 h; **C_max_** = maximum drug concentration in plasma; **t_1/2_** = terminal elimination half-life.
