# Supplementary material for: Volume replacement strategies do not impair the binding of dabigatran to idarucizumab: Porcine model of hemodilution
Source: PLoS One. 2019 Jan 7;14(1):e0209350. doi: 10.1371/journal.pone.0209350 (PMC6322768; doi:10.1371/journal.pone.0209350)
Supplement: S3 Table — Results are presented as mean (± SD), n = 5/group, unless stated otherwise. (DOCX) [file pone.0209350.s003.docx]

**S3 Table** Summary pharmacokinetic parameters of idarucizumab in pigs after intravenous dosing^a^

| **Group** | **C_max_ (nM)** | **AUC_0-24_ (nM·h)** | **CL (mL/min/kg)** | **t_1/2_ (h)** |
| --- | --- | --- | --- | --- |
| Control | 8610 ± 1310 | 13920 ± 1583 | 0.758 ± 0.088 | 3.17 ± 0.22 |
| Gelatin | 7460 ± 880 | 15460 ± 1462 | 0.681 ± 0.073 | 3.16 ± 0.38 |
| 6% HES 200/0.5 | 6540 ± 499 | 14520 ± 1287 | 0.724 ± 0.070 | 2.84 ± 0.42 |
| Ringer’s Solution | 8520 ± 170 | 16860 ± 2889 | 0.632 ± 0.101 | 3.59 ± 0.52 |
| 6% HES 130/0.4 | 5940 ± 429 | 12100 ± 1310 | 0.873 ± 0.089 | 3.38 ± 0.80 |
| **All combined** | 7830 ± 1730 | 14572 ± 1772 | 0.721 ± 0.118 | 3.23 ± 0.28 |

^a^Results are presented as mean (± SD), n=5/group.

**AUC_0-24_** = area under the drug plasma concentration-time curve for time 0 to 24 h; **C_max_** = maximum drug concentration

in plasma; **t_1/2_** = terminal elimination half-life.
